# Index Medicus

**Published:** 1885-05

**Authors:** 


### INDEX MEDICUS.

It will be welcome news to many that Geo. S. Davis, the well-known medical publisher of Detroit, will henceforth be the publisher of *Index Medicus.* That journal, though a very important one in medicine, has never paid its way, and it is a gratifying evidence of public spirit when a responsible man steps into the breach, and interferes to save such a meritorious work from being blotted out. It is to be hoped that Mr. Davis will not, in the end, be a loser by his enterprise.

